# Longitudinal analysis of antibody responses in symptomatic malaria cases do not mirror parasite transmission in peri-urban area of Cote d’Ivoire between 2010 and 2013

**DOI:** 10.1371/journal.pone.0172899

**Published:** 2017-02-28

**Authors:** David Koffi, Marie-Louise Varela, Cheikh Loucoubar, Sylvain Beourou, Inès Vigan-Womas, Aissatou Touré, Joseph Allico Djaman, André Offianan Touré, Ronald Perraut

**Affiliations:** 1 Institut Pasteur de Côte d’Ivoire, Unité de Paludologie, Abidjan, Côte d’Ivoire; 2 Université Félix Houphouet Boigny, UFR Biosciences, Abidjan, Côte d’Ivoire; 3 Institut Pasteur de Dakar, Unité d’Immunologie, Dakar, Sénégal; 4 Institut Pasteur de Dakar, G4 Biostatistiques Bioinformatique et Modélisation, Dakar, Sénégal; 5 Institut Pasteur, Unité d’Immunologie Moléculaire des Parasites, Paris, France; 6 Institut Pasteur de Madagascar, Unité d’Immunologie, Antanarivo, Madagascar; Université Pierre et Marie Curie, FRANCE

## Abstract

**Background:**

In the agenda towards malaria eradication, assessment of both malaria exposure and efficacy of anti-vectorial and therapeutic strategies is a key component of management and the follow-up of field interventions. The simultaneous use of several antigens (Ags) as serological markers has the potential for accurate evaluation of malaria exposure. Here we aimed to measure the longitudinal evolution of the background levels of immunity in an urban setting in confirmed clinical cases of malaria.

**Methods:**

A retrospective serological cross-sectional study on was carried out using 234 samples taken from 2010 to 2013 in peri-urban sentinel facility of Cote d’Ivoire. Antibody responses to recombinant proteins or BSA-peptides, 8 *Plasmodium falciparum* (PfAMA1, PfMSP4, PfMSP1, PfEMP1-DBL1α1-PF13, PfLSA1-41, PfLSA3-NR2, PfGLURP and PfCSP), one *P*. *malariae* (PmCSP) and one *Anopheles gambiae* salivary (gSG6-P1) antigens were measured using magnetic bead-based multiplex immunoassay (MBA). Total anti- *P*. *falciparum* IgG responses against schizont lysate from african 07/03 strain (adapted to culture) and 3D7 strain was measured by ELISA.

**Results:**

High prevalence (7–93%) and levels of antibody responses to most of the antigens were evidenced. However, analysis showed only marginal decreasing trend of Ab responses from 2010 to 2013 that did not parallel the reduction of clinical malaria prevalence following the implementation of intervention in this area. There was a significant inverse correlation between Ab responses and parasitaemia (P<10^−3^, rho = 0.3). The particular recruitment of asymptomatic individuals in 2011 underlined a high background level of immunity almost equivalent to symptomatic patients, possibly obscuring observable yearly variations.

**Conclusion:**

The use of cross-sectional clinical malaria surveys and MBA can help to identify endemic sites where control measures have unequal impact providing relevant information about population immunity and possible decrease of transmission. However, when immunity is substantially boosted despite observable clinical decline, a larger cohort including asymptomatic recruitment is needed to monitor the impact of control measures on level of immunity.

## Introduction

*Plasmodium falciparum* malaria remains a major threat in tropical and sub-tropical regions, with nearly 50% of the world population exposed to infective bites by Anopheles mosquitoes and almost half million deaths annually [1]. Scaling up of integrated interventions strategies including artemisinin-based combination therapy (ACT), universal coverage with long-lasting insecticide-impregnated bed nets (LLINs), systematic diagnosis using rapid tests (RDTs) and intermittent preventive treatment in vulnerable target groups have considerably reduced the burden of malaria in many countries and saved more than a million lives since the year 2000, most of them among children under 5 years of age [2].

Presently, the number of malaria cases is still very high (more than 214 million malaria cases) as well as the number of deaths (236 000–635 000 according to the WHO 2015). Furthermore, in addition to the threats associated with the emergence of resistance to artemisinin in Southeast Asia and insecticides in Africa, malaria rebound in some countries like Rwanda, Sao Tome, Principe and Zambia that were the leaders in the early upgrade of fighting efforts [2].

Monitoring changes in malaria transmission intensity and disease prevalence through surveillance allows health authorities to evaluate health services and plan control programs.

Sero-surveillance is based on the use of *Plasmodium* species-specific antibodies as indicators for exposure, transmission, and immunity. Such tool has a significant potential for contributing to the effectiveness of malaria control and elimination program. Antibodies are very sensitive marker of malaria exposure in low-transmission settings and reflect cumulative exposure over a period of time, which is useful in areas with highly seasonal transmission [3,4]. Although this approach was used historically as part of malaria control programs, its use was not developed in part because of the lack of standardized antigens and methodology [4]. Of more than 5,000 proteins expressed by the Plasmodium species, few have been examined in any detail, and there is a trend towards further development of sero-epidemiological analysis for monitoring malaria control and elimination. A comprehensive evaluation of candidate antigens as biomarkers is required to identify those antibody responses that are most sensitive for detecting changes in transmission. Studies employing protein microarrays [5] or expanded repertoires of purified antigens [6] are beginning to address this knowledge gap, and it is likely that multiple antigens will need to be included in serologic assays [4,5,6,7].

Several teams use sero-epidemiology analysis in low transmission settings focusing investigations on change of seroprevalence levels. They use results from cross-sectional studies to build mathematical seroconversion rate models and predict decline of malaria transmission [8].

In addition, few analyses were focused on symptomatic cases. Parasite invasion and multiplication in human strongly stimulates immune responses leading to possible higher individual antibody responses in more exposed persons that thereby possess a higher degree of acquired immunity [9,10]. Thus, Ab responses measured during symptomatic episode rather represent surrogates of an effective immune background depending upon duration and intensity of parasitemia before treatment. Cross sectional analysis of randomly recruited symptomatic and well documented cases from sentinel sites in Cote d’Ivoire, showed that multi-target measure of Ab responses could represent surrogates of actual immune background [11].

In the present study, we used a similar approach in a longitudinal manner by recruiting individuals living in Abobo from 2010 to 2013, a peri-urban setting of Côte d’Ivoire with high continuous parasite transmission. We aimed to verify here if previous observation in different settings was available in the same setting where an increased efficient malaria control program was applied from 2010 to 2013. Antibody responses against 12 antigens targets was analyzed using a magnetic bead-based multiplex assay [12] together with IgG responses to whole parasite extract with ELISA. Results showed that the profile of antibody responses did not reflect the evolution of the decreasing transmission resulting from increased prevention measures and actions at community level.

## Material and methods

### Study area, procedures for recruitment

Subjects were recruited in Abobo, a periurban Ivorian malaria endemic area included in the Sentinel National Network for Surveillance of Malaria. The site of Abobo is located in the southern part of the township of Abidjan, characterized by the presence of the laguna and a parasite transmission occurring all year round.

The study was conducted in accordance with the local laws and regulations, International Conference on Harmonization—Good Clinical Practice (ICH-GCP). The protocol was reviewed and approved by the Comité National d’Ethique et de Recherche de Côte d’Ivoire (N°56/MSLS/CNER-dkn). Individual written informed consent was obtained from participants/ parents/ guardians. In case of an illiterate patient, his/her thumb impression and signature of an independent witness were obtained.

There was a particular exception in 2011 where young asymptomatic individuals were recruited in a transversal survey. This particular survey was done under request and ethical approbation from the Ministry of health (N°01/MSHP /064) as routine recruitment was not possible at the hospital because of the civil war. The parents or guardians and school administrator were duly informed of the objectives and methodology of this survey, they agreed to participate to the study and provided useful information about their children. This survey involved 207 volunteer school children (6 to 15 years old) with normal axillary temperature <37.5°C. They were examined for parasite positivity: 59 individuals were positive by RDT including 35 individuals positive by blood smear.

The overall study involved 234 individuals, 175 patients consulting for symptomatic fever in healthcare center formation sanitaire Anonkoua-Kouté in Abobo, and 59 schoolchildren in 2011. Symptomatic patients were treated and followed-up according to the standard national procedure. Diagnosis of malaria includes RDT test, blood sampling for biological investigations and blood smear for parasite counting. Parasitemia was counted on thick blood smears by two experienced microscopists. In case of discrepancy, smears were confirmed by a third counting. Characteristics of the followed groups are summarized in Table 1. All consultants were treated with ACT, according to the national therapeutic policy. They were hospitalized, treated and followed-up daily from day 0 to day 3 in the healthcare center. Parasitaemia were recorded every 24 hours up to two consecutive negative blood smear of patient. The parasite clearance time (PCT) is recorded for each patient, an indicator defined as the time between treatment and the first negative slide.

**Table 1:** Context and characteristics of the study population.

Year of recruitment	2010	2011	2012	2013
Month of sampling	October	June	August	September
No of individuals	57	35 24*	56	62
Proportion M/F (%)	26/31 (46%)	31/28 (53%)	32/24 (57%)	24/38 (39%)
Age [median min-Max]	8.0 [2–12]	11.0 [6–16] 11.0 [9–13]*	12.5 [1–52]	13.0 [2–68]
Hemoglobin mean (SD)	9.8 (1.9)	11.8 (1.3)	11.0 (2.0)	10.7 (1.7)
% of anemia ≤ 9 g/dL	32%	0%	14%	21%
Platelets mean (SD)	127 (64)	232 (84)	162 (122)	148 (73)
Mean parasitemia (parasitemia SD)	90213 (100278)	8103 (19598)	34942 (28473)	61731 (88462)
Parasitemia in different age-groups				
≤ 5 years old.	115518	na	51737	85555
[min-max]	[1331–341700]	na	[6000–99860]	[2300–200000]
6–10 years old.	91269	2892	39937	74604
[min-max]	[1845–336830]	[0–20520]	[7920–97340]	[2180–198600]
11–15 years old.	54926	11182	41460	87268
[min-max]	[1764–174879]	[0–106800]	[6400–86340]	[7894–587000]
> 15 years old.	na	na	21470	28235
[min-max]	na	na	[1160–86280]	[2100–84307]

* individuals from asymptomatic recruitment in 2011 with negative RDT

For all patients, almost complete parasite clearance within 3 days was observed after treatment. In 2012 and 2013, 88% and 55% of treated individuals cleared their parasitemia in 24h, respectively.

Plasma samples were collected after biology processing at day 0 upon centrifugation, and stored at -20°C until further analysis.

No entomological data were available for the area of Abobo. However, in the absence of exact EIR, overall morbidity data collected from health facility records in 2013 signifies the high level of transmission in this endemic area.

### Antigens and peptides

Three soluble recombinant proteins and 8 peptides conjugated to BSA (Bovine Serum Albumin) specific to *P*. *falciparum*, *P*. *malariae* and *Anopheles gambiae* salivary peptide gSG6 antigen (Ag) were included. BSA provided by peptide manufacturer was used as carrier control. The peptides used in our studies were designed as already described [13], a N-terminal cysteine residue was added to allow a unidirectional coupling to BSA by the manufacturer (GenScript HK Inc.,Hong Kong, China, or Genecust, France). Purity of each BSA-peptide was estimated >85% by HPLC and mass spectrometry. A summary of antigens and peptides used is given in Table 2.

**Table 2:** Summary of antigens used for measurement of Ab responses.

Antigen	Protein or peptides	Antigen from	Stage of expression^1^	Sequence and/or Reference
CSP	Peptide	*P*. *falciparum*	*sporozoite/hepatic stage*	NANPNANPNANPNANPNANPNANPNANPNANPNANPNVDPNVDPC
LSA1_41_	Peptide	*P*. *falciparum*	*intra-hepatocyte*	LAKEKLQEQQSDLEQERLAKEKLQEQQSDLEQERLAKEKEKLQC
LSA3	Peptide	*P*. *falciparum*	*blood stages*	VLEESQVNDDIFNSLVKSVQQEQQHNVC
*SALSA*	Peptide	*P*. *falciparum*	*merozoite*	SAEKKDEKEASEQGEESHKKENSQESAC
GLURP	Peptide	*P*. *falciparum*	*merozoite/schizont*	EDKNEKGQHEIVEVEEILC
AMA1	Peptide	*P*. *falciparum*	*sporozoite/merozoite*	YKDEIKKEIERESKRIKLNDNDDEGNKKIIAPRIFISDDKDSLKC
PF13	Protein	*P*. *falciparum*	*mature blood stages*	DBL1 domain of 3D7-PF13_0003 var gene
MSP1p19	Protein	*P*. *falciparum*	*merozoite*	Bonnet et al, Vaccine 2006
MSP4p20	Protein	*P*. *falciparum*	*merozoite*	Bonnet et al, Vaccine 2006
*Pm*CSP	Peptide	*P*. *malariae*	*sporozoite*	NAAGNAAGNAAGNAAGNAAGNAAGNAAGNAAGNAAGNAAGNDAGC
gSG6	Peptide	*A*. *gambiae*	*Salivary gland*	EKVWVDRDNVYCGHLDCTRVATFC
BSA	-	control		

^1^ pre-erythrocytic and/or erythrocytic localization of Ag tested (from www.plasmodb.org)

The procedure for production and purification of the NTS-DBL1α1 domain of the PfEMP1 (*P*. *falciparum* Erythrocyte Membrane Protein-1) adhesin encoded by the 3D7/PF13 var gene has been reported elsewhere [14]. Soluble recombinant protein corresponding to *P*. *falciparum* MSP1p19 and MSP4p20 were produced in the baculovirus / insect cell expression system and purified by metallo-affinity chromatography as described [15,16].

### Coupling of Ag to beads

The covalent coupling of recombinants antigens (PF13, MSP4p20 and MSP1p19), BSA and all the 8 peptides to carboxylated magnetic Luminex microspheres was done by the carbodiimide reaction (Luminex corp, Austin, USA) using the xMAP^®^ Antibody Coupling Kit following manufacturers’ instructions (ref 40–50016, Luminex corp, Austin, USA).

Briefly, 2.5x10^6^ beads from regions 26 to 39 and 5 μg Ag per million beads in a working volume of 500μL were used. All steps of washings, buffer changing after 1–2 min centrifugation at 8000 x g, magnetic pelletting using the Luminex^®^ Magnetic plate separator (Luminex corp, Austin, USA), vortexing and sonication in water-bath sonicator to disperse the beads, carbodiimide hypochloride (EDC) activation, conjugation under rotation mixing and final pelletting of conjugated beads was done as already described [11,12,17]. Final count of remaining beads using cell counter showed a mean recovery of 96% of the coupled beads. Efficient coupling of Ag was controlled using positive individual and pool of human sera. The coupled microspheres were kept in the washing/ storage buffer at 4°C in the dark until use.

### Bead-based assay for IgG antibodies

The Magnetic Bead-based MAGPIX^®^-Luminex Assay (MBA) was used to parallel the working steps used in the standard ELISA technique as already described [11,12,17]. 2.5 μL aliquots of the mix of microspheres containing 3000 beads per Ag were distributed to individual wells of a white polystyrene opaque round bottom microtiter plate (Ref.103977741, Fisher Scientific, Illkirch, France). 100 μL plasma diluted 1:100 in PBS Tween 0.01% BSA 1% (PBSB) was added in duplicate wells, mixed and incubated with the beads protected from light on a microplate shaker. After removal of plasma and two washing steps with 100μL PBSB, 100μL phycoerythrin-labeled goat anti-human IgG diluted 1:500 (gamma- chain specific, F(ab`)_2_ fragment-R-phycoerythrin (Sigma, P-8047 St. Louis, MO) in PBSB was added and incubated in the dark with shaking. The beads were finally resuspended in 120 μL PBSB after two washes and analyzed on a Multiplex MAGPIX system (Millipore, USA) using the xPONENT 4.1 software for acquisition. Antibody responses were expressed in median fluorescence intensity (MFI) per sample as stated by manufacturer’s instructions; individual positivity was considered when the signal was greater than 2 x [mean MFI signal + 3 SD of 6 naïve control sera].

### ELISA procedure

IgG responses to whole parasite extract Ag using 07/03 Dielmo strain were quantified by ELISA in duplicate plasma samples diluted 1:100 as previously described [18,19,20]. Positive and negative controls were included in each assay *ie* a pool of 25 sera from clinically immune adults living in the village of Dielmo (a holoendemic area of transmission in Senegal) and a pool of European and/or African non-immune, respectively. Results were expressed as OD ratio = OD sample / OD naive serum pool [19,21]. Sera showing an OD ratio >2 (corresponding to the signal of naive controls + 3 SD) were considered sero-positive for prevalence analysis.

### Statistical analysis

Antibody levels and prevalence of responders in different groups were compared using the Mann-Whitney signed rank test, the Spearman rank correlation test for non-normally distributed paired data and the fisher exact test. P values <0.05 were considered significant. Statistical analyses were performed with R and Statview 5.0 (SAS Institute) software.

To study the correlation between level of parasitaemia and levels of antibody responses a log transformation has been used. The log-transformed data were shown distributed according to normal law using Shapiro test. The log-transformed were included in a generalized linear model adjusted for age to analyse distribution of parasitaemia level—considered as dependent variable—as function of antibody responses to different targets.

## Results

### Indicators for effective field application of malaria prevention measures

**Fig 1:**
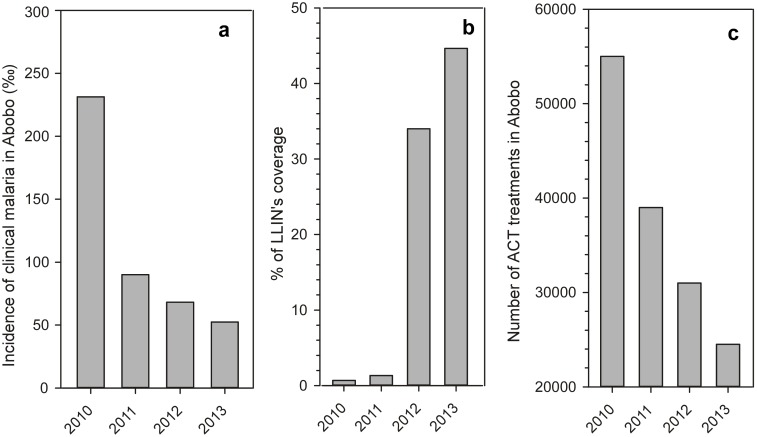
Indicators of malaria prevention measures in Abobo from 2010 to 2013. Longitudinal annual profiles of incidence of clinical malaria (a), percentage of population coverage by LLIN distribution (b) and number of ACT treatments distributed (c) are shown as histogram plots. These indicators illustrate the substantial increase of prevention measures from 2010 to 2013 (data from RASS 2013—Rapport Annuel des Service de Sante, Ministère de la Sante et de la Lutte contre le SIDA, Côte Ivoire).

As illustrated on Fig 1, there was a strong increase of prevention measures against malaria from 2010 to 2013 in Abobo with highly significant results. There was a substantial decrease of clinical malaria reports falling of 77% from 231‰ to 52‰ (Fig 1a), an observation that paralleled a 55% decrease of distribution and use of ACT treatments (Fig 1b). Indeed, prevention measures were accompanied by a strong implementation of distribution of impregnated bednets (Fig 1c) for coverage of population. In 2013, the % of coverage by LLIN’s reached 44.6% with an overall ongoing objective of 60% of coverage for the population in Abobo, a goal almost reached in 2016. All data are from Ministry of Health (RASS 2013—Rapport Annuel des Service de Sante, Ministère de la Sante et de la Lutte contre le SIDA, Côte Ivoire)

### Characteristics of the cohort

Recruitment was done during the rainy season (from May to November). As shown in Table 1, overall mean age was low 13.5 (median = 11; 1–68 years) with variable ranges from 2010 to 2013. Of the four groups, one was significantly younger than the others *ie* in 2010, because of the heterogeneity from the available serum biobanks. To overcome such heterogeneity for analysis, individuals were categorized in 4 age-groups for further comparisons ie ≤5; 6–10; 11–15; >15 years old (see Table 1).

As shown in Table 1, levels of parasitemia measured upon recruitment were highly variable, with significant lower level in 2011 compared to the other years (P<0.001). As a matter of fact, cross sectional recruitment from 2011 was different, involving asymptomatic individuals. Interestingly, in these individuals parasite carriage was far from negligible: 27% had positive RDT, 16% had a positive blood smear. In the set of sample tested here, median parasitemia reached 3200 trophozoite per μl.

There was a significant relationship between parasitemia (or log of parasitemia) and age of symptomatic individuals sampled in 2010, 2012 and 2013 (P<0.001, rho = -0.30), but not in 2011.

### Mean prevalence and levels of antibody responses

As summarized in Table 3, mean prevalence levels of responders were variable depending upon Ags rather than year of analysis, ranging from 2.9% (IgG to gSG6 peptide in 2011) to 97.1% (IgG to MSP4p20 and schizont extract in 2011). There was no significant difference for prevalence levels between the different years for any Ag. Level of prevalence was generally homogeneous for a given Ag, allowing stratification in 3 levels: (i) Ags strongly recognized >70% positivity (schizont extract, SALSA, PF13, MSP1p19, MSP4p20); (ii) an intermediate recognition level [70%-25%] for CSP, LSA1_41_, GLURP and AMA1 antigens and; (iii) Ags weakly recognized <25% positivity (LSA3, PmCSP, gSG6).

**Table 3:** Mean levels and prevalence of IgG Ab responses to the panel of antigens.

Antigens	2010		2011				2012		2013	
	Mean Ab level	Prevalence	Mean^1^ Ab level	Prevalence^1^	Mean^2^ Ab level	Prevalence^2^	Mean Ab level	Prevalence	Mean Ab level	Prevalence
Schiz. Extr.	3,4	92.9%	3,7	97.1%	3,1	91.7%	3,3	87.5%	3,4	88.5%
CSP	109	40.4%	79	20.0%	90	33.3%	148	32.1%	126	41.9%
LSA1_41_	349	64.9%	177	25.7%	262	41.7%	281	48.2%	299	53.2%
LSA3	83	12.3%	78	8.6%	53	4.2%	72	5.4%	67	6.5%
*SALSA*	413	80.7%	1354	88.6%	1041	70.8%	1273	76.8%	993	71.0%
GLURP	219	49.1%	417	80.0%	294	62.5%	361	57.1%	345	66.1%
AMA1	208	56.1%	257	57.1%	246	41.7%	292	50.0%	341	40.3%
PF13	1220	93.0%	2291	94.3%	2189	83.3%	1792	89.3%	1759	82.3%
MSP1p19	3346	93.0%	2347	88.6%	1670	100%	3485	94.6%	3131	96.8%
MSP4p20	2269	93.0%	3371	97.1%	2839	91.7%	2605	91.1%	1872	93.5%
*Pm*CSP	136	7.0%	269	25.7%	293	29.2%	138	8.9%	125	8.1%
gSG6	58	29.8%	34	2.9%	35	8.3%	40	8.9%	38	6.5%

^1^Mean antibody levels and prevalence of individuals RDT positive in 2011

^2^Mean antibody levels and prevalence of individuals RDT negative in 2011

Regarding mean levels of Ab responses, excluding year 2011, almost Ab responses did not differ significantly from 2010 to 2013.

Importantly, asymptomatic individuals recruited in 2011 showed high Ab levels comparable with those of symptomatic patients from years 2012 and 2013. When comparing 2010 to 2011, some significant differences (P<0.05) were found: lower levels for CSP, LSA1_41_, LSA3, higher for SALSA, GLURP, PF13 and MSP4p20.

### Relationship between Ab responses and circulating parasitemia

The analysis of relationship between Ab levels and circulating parasitemia for symptomatic patients on years 2010, 2012 and 2013, showed a significant negative relationship for schizont extract, CSP and LSA1_41_ Ags (spearman test, P<0.005, rho # -0.25). The use of log transformation for parasitemia data led to a similar significant relationship with the same Ag targets (spearman test, P<10^−3^, rho # -0.21).

Further analysis was done by stratifying levels of parasitemia in 4 classes: (i) positive [<10^4^]; (ii) elevated [10^4^–5x10^4^]; (iii) high [5x10^4^–10^5^]; (iv) very high [>10^5^] trophozoite per μl. Levels of Ab responses were not significantly different as function of levels of parasitemia for any Ag in 2010. Analysis of 2012 cohort showed significant high Ab responses against CSP and LSA1_41_ (P<0.01) in individual with the lowest level of parasitemia compared to the highest one (<10^4^ *vs* 5x10^4^-10^5^ trophozoite per μl). For patients recruited in 2013, similar result was found for Ab responses against schizont extract Ag (P<0.01).

**Fig 2:**
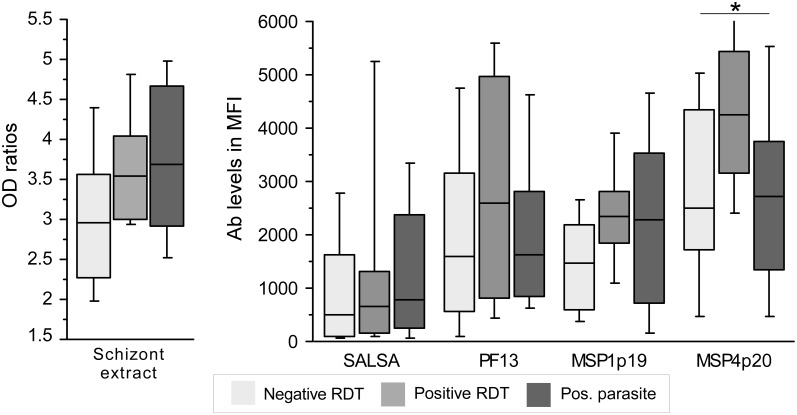
Ab responses as function of presence of circulating parasites in asymptomatic group recruited in 2011. Levels of Ab responses against schizont extract, SALSA, PF13, MSP4p20 and MSP1p19 are illustrated as boxplot for asymptomatic individuals recruited in 2011 as function of their parasite carriage *ie* RDT negative, RDT positive, RDT and blood smear positivity. Significant differences (Kruskal wallis test, P<0.05) are indicated by an asterisk.

Analysis for the group of individuals recruited in 2011 was done after stratification according to RDT: negative, positive with/without circulating parasite. As illustrated on Fig 2 (plotting of Ab responses to schizont extract and Ags strongly recognized), there was a trend for higher responses in RDT positive individuals and a slight decrease when circulating parasite was detectable by blood smear. Significant different level were found only for MSP4p20 (P = 0.048).

Multiple linear regression adjusted on age-groups including all years but 2011 showed no Ab responses significantly (P<0.05) associated with lower parasitemia level (just reaching significant for schizont extract and CSP, P = 0.05).

When analyzing year 2011 only, Ab response to GLURP were significantly associated with lower parasitemia level (P = 0.03).

When regression model included all years and was adjusted on age and years of follow-up, Ag targets associated with lower parasitemia were: GLURP (P = 0.03), MSP4p20 and PF13 (P = 0.01).

### Age-groups associated longitudinal comparison of antibody levels in 2010, 2012 and 2013

Analysis of interrelation between antibody responses and age of individuals showed significant positive correlations (P<0.01, Spearman rank test) for schizont extract CSP, SALSA, GLURP and PF13 Ags (rho from 0.2 to 0.3).

**Fig 3:**
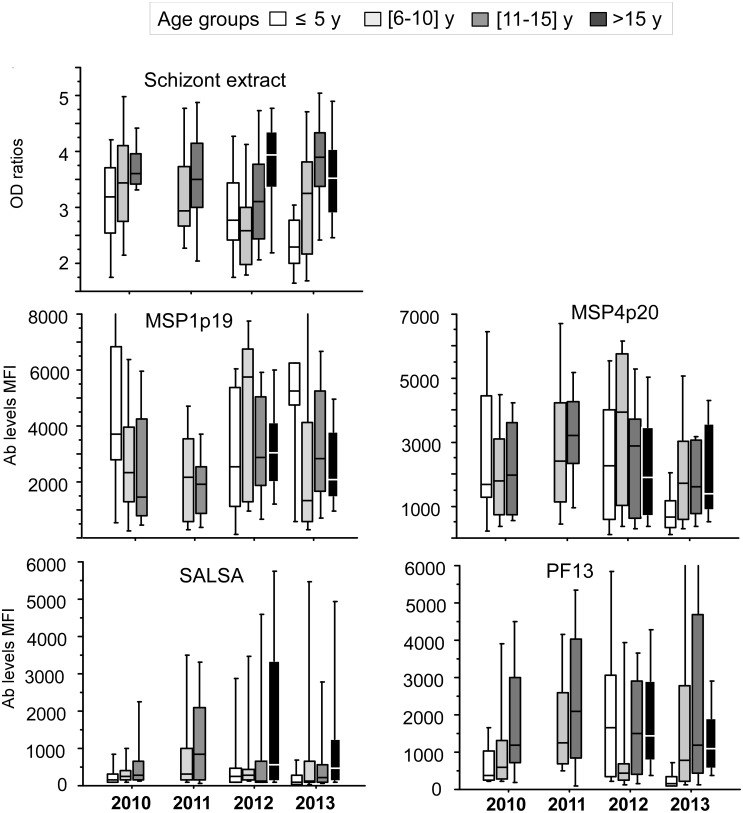
Ab responses against strongly recognized *P*.*falciparum* antigens in age-groups from 2010 to 2013. Profiles of antibody responses measured by ELISA or multiplex magnetic bead based fluorescence assay are plotted as mean OD ratio or Median fluorescence levels from 2010 to 2013 for individuals from 4 different age groups: < 5 years old (empty), 6-10yo (light grey), 11–15 years old (dark grey), >15 years old (black). Schizont extract and antigens strongly recognized are shown ie MSPs, SALSA and PF13.

**Fig 4:**
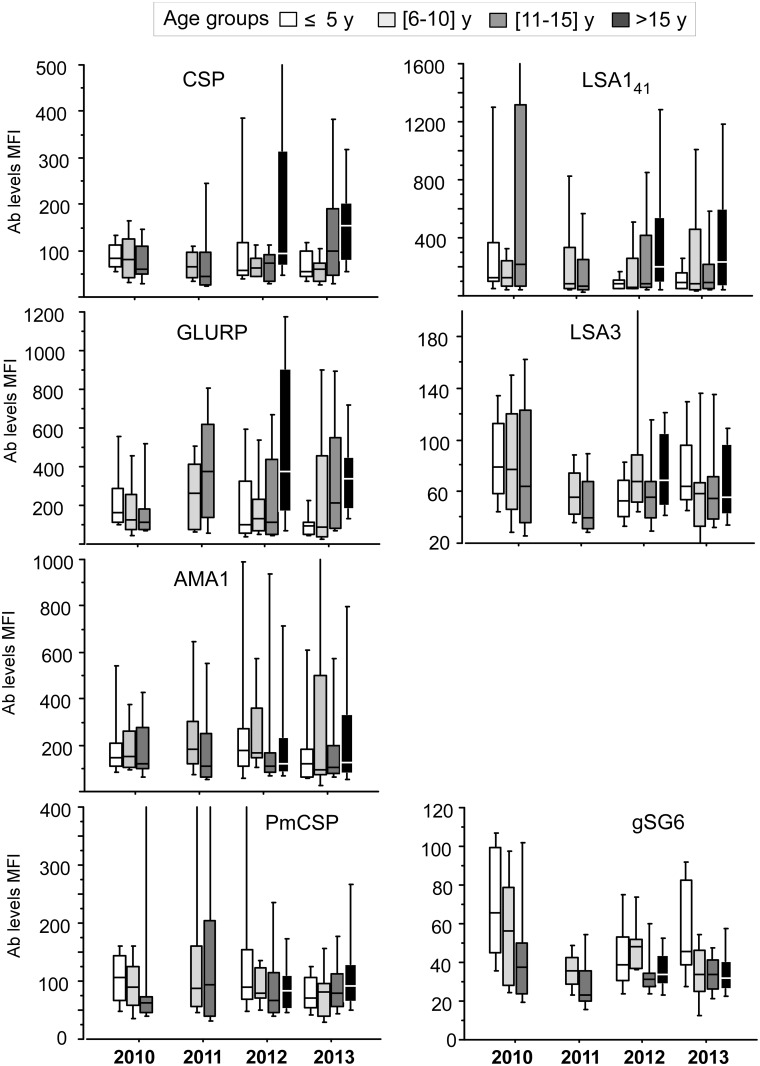
Age-group profiles of Ab responses against *Plasmodium* and *A*. *gambiae* antigens from 2010 to 2013. Antibody responses measured by magnetic bead based fluorescence assay are plotted as Median fluorescence levels from 2010 to 2013 for individuals from 4 different age groups: < 5 years old (empty), 6-10yo (light grey), 11–15 years old (dark grey), >15 years old (black). On this figure are plotted antibody responses to *P*.*falciparum* Ags tested not shown on Fig 3 together with *P*. *malariae* CSP and *A gambiae* salivary peptide gSG6.

Profiles of Ab responses by age-groups are shown on Figs 3 and 4. The age-related increasing profile of Ab responses from symptomatic patients regarding 5 strongly recognized Ag tested (SE, MSPs, SALSA and PF13) are observable on Fig 3, the profiles from the 7 other Ags are shown on Fig 4. A very limited year-associated decline of Ab responses from 2010 to 2103 was evidenced. Trend test from 2010 to 2013 (without 2011) adjusted on age groups showed significant trend for decrease only for Abs levels against gSG6 (P<0.001)

Results from systematic year by year dichotomy comparisons (Mann Whitney test) are summarized in Table 4. The limited trend to decline when analyzing years 2010–2012 and 2013 was confirmed statistically. Only a slight decline was significant for PF13 Ag in younger age groups. Marginal significant decreases were evidenced in younger age-group for LSA1_41_, LSA3 and gSG6 salivary peptide.

**Table 4:** Systematic comparison year by year of levels of IgG Ab responses in age-stratified groups.

	2010 vs 2012				2010 vs 2013				2012 vs 2013				
	All	<6y	6_10y	11_15y	All	<6y	6_10y	11_15y	All	<6y	6_10y	11_15y	>15y
Schiz. Extrc.	ns	ns	0.01	ns	ns	ns	ns	ns	ns	ns	ns	ns	ns
CSP	ns	ns	ns	ns	ns	ns	ns	ns	ns	ns	ns	ns	ns
LSA1_41_	ns	0.03	ns	ns	ns	ns	ns	ns	ns	ns	ns	ns	ns
LSA3	ns	0.02	ns	ns	0.03	ns	ns	ns	ns	ns	ns	ns	ns
*SALSA*	ns	ns	ns	ns	ns	ns	ns	ns	ns	ns	ns	ns	ns
GLURP	ns	ns	ns	ns	ns	ns	ns	ns	ns	ns	ns	ns	ns
AMA1	ns	ns	ns	ns	ns	ns	ns	ns	ns	ns	ns	ns	ns
PF13	0.03	0.04	ns	ns	ns	0.01	ns	ns	ns	0.01	ns	ns	ns
MSP1p19	ns	ns	ns	ns	ns	ns	ns	ns	ns	ns	ns	ns	ns
MSP4p20	ns	ns	ns	ns	ns	ns	ns	ns	ns	ns	ns	ns	ns
PmCSP	ns	ns	ns	ns	ns	ns	ns	ns	ns	ns	ns	ns	ns
gSG6	0.003	0.01	ns	ns	ns	ns	0.04	ns	ns	ns	0.04	ns	ns

## Discussion

There is a high level of malaria endemicity in Côte d’Ivoire [1] and a considerable heterogeneity in the geographic distribution of transmission intensity as function of climatic variation from north to south, and environmental factors including human activities [22,23,24]. National surveillance system reported an increase of presumed and clinical cases from 2.8 to 4.7 million in 2012 and 2013, respectively [1] highlighting the strengthening of the surveillance and recording process. However, malaria remains a major threat, responsible of 43% of the consultations in health center, with an important impact on children under 5 year old and pregnant women. Consequently, malaria has a strong socio-economic impact, responsible for 40% and 42% of school and working absenteeism, respectively. Families spend about 25% of their income for the treatment and prevention of malaria.

The scaling up of integrated interventions strategies, as reported for Abobo, showed clearly a substantial positive result between 2010 and 2013 in Abobo (Fig 1), underlining the necessity to increase monitoring and evaluation for close follow-up of elimination strategy.

A pilot study conducted in 3 different settings of Côte d’Ivoire highlighted the potential of *Plasmodium* species-specific antibodies as indicators for exposure when analyzing multi-target Ab responses in acute malaria patients from sentinel health center. Malaria transmission in these settings including Abobo was high; profile of antibody responses was found different according to the location of the setting, accurately reflecting the immune background [11]. A similar approach has been tested in the present study to investigate the longitudinal profile of antibody responses in the peri-urban sentinel setting of Abobo where malaria burden strongly decreased between 2010 and 2013.

Despite the observed decrease of malaria burden, analysis of the antibody responses to such array of antigens in this study could not sustain any substantial decrease of immune background level in those patients. Indeed, antibody responses were high as expected in individuals where parasite invasion induced clinical symptoms. The age-related significant increase of antibody responses found here witnesses a profile of a site with high ongoing endemic transmission, whose actual decrease is not yet reflected by detectable variation of the immune background. Several factors can contribute to this observation: firstly the recruitment of symptomatic individuals with high antibody boosting, secondly the short time frame of only two years between the strong implementation of prevention measures conducted in 2010–2011 and the latest measure in 2013 and thirdly the lack of biomarkers adapted to precise exposure estimates [25].

Studies using serological analyses as indicators of malaria transmission generally involve recruitments in a relatively stable state for host-parasite interaction, usually of asymptomatic individuals in different settings and/or in longitudinal manner. Several antigens have been used as markers of exposure and malaria risk [9,26], including comparable multiplex antigen arrays [13,27]. Reducing the number of antigens as biomarkers would be an optimal solution as described by Cook and al using a single antigen such AMA1 for mapping effectiveness of control intervention in an island environment of Guinea [28]. As a general matter, several antigens are required at least for analyzing biomarkers of immunity that may probably differ from those used as biomarkers of exposure [25]. Several studies involved MSP1p19, AMA-1 and MSP2 as serological markers for medium and long-term indicator of malaria transmission in settings with various altitudes [29] or mapping heterogeneity of transmission in low endemic places [30]. However, until now, there are no clear correlates of anti-malarial immunity with antibody responses against a given target.

Numerous results and data underline antibodies to recombinant merozoite antigens as valuable biomarkers of immunity [6,31]. Therefore among these antigens, MSP1p19 is an interesting target for exposure and protection. It has been used as a relevant tool for longitudinal retrospective sero-surveillance in the Gambia providing valuable information about population immunity as well as exposure, as transmission declines and immunity wanes [8]. The baculovirus expressed recombinant MSP1p19 used here, with relevant conformational structure [32,33], was shown to correlate with protection [20] and had good concordance with ELISA measures when used coated on beads [12].

Altogether, analysis of antibody responses in case of longitudinal symptomatic malaria recruitment would rather require testing a multiplex array of antigens as a potential valuable tool. Antibody responses to different parasite targets and other plasmodium species were useful: in this study, IgG response to *Anopheles* salivary peptide gSG6-P1 antigen was the only indicator of a significant trend to decrease, a relevant marker of risk for malaria transmission already underlined [34,35].

Regarding parasitemia, no trend of differential susceptibility was evidenced in the different groups. Levels of parasitemia were comparable (except in asymptomatic individuals from 2011), in line with the almost similar levels of immunity measured in 2010, 2012 and 2013. Interestingly, young individuals harbored higher parasitemia and a significant relationship between high levels of Ab to GLURP, PF13 and MSP4p20 and lower parasitaemia was found. These results confirm that antimalarial antibodies may play an important supportive role in the therapeutic response to antimalarial drugs during acute falciparum malaria as already observed [36,37,38]. In 2013, the % of individual clearing their parasitemia in 24h was lower than in 2012 (55% *vs* 88%, P<10^−3^ by fisher exact test). Whether this observation relates with a lower efficient level of immunity for parasite clearance requires further longitudinal investigation on a larger cohort of individuals.

Taken together, this study lacked of power for analyzing potential decrease of immunity expected to parallel the observable decrease of malaria burden due to effectiveness of control measures. However, this study underlined several interesting information regarding analysis of immune responses in high endemic setting. The 2011 particular recruitment shows clearly that immune background and natural immunity are substantially high. A proportion of 27% of young schoolchildren had positive RDT, in these individuals 16% had circulating parasitemia over 10^5^ trophozoite per micoliter of blood without any clinical symptoms. In these children, elevated antibody levels and prevalence against the array of antigens were found reaching higher levels than symptomatic individuals for SALSA, GLURP, PF13 and MSP4p20. In non-parasitized RDT positive individuals, there was a trend towards highest levels of antibody (Fig 3), indicating a background of robust induction of antibody responses and a persistence of antibodies from earlier infections without clinical manifestations [10]. It is likely that strong induction and boosting of antibody responses might occur in Abobo, which might have obscured the baseline antibody levels and consequently hiding the expected decrease of immune levels in symptomatic individuals as it has been observed in different settings [11]. Further investigations are needed, targeting a wider scale recruitment of population by including simultaneously cross sectional survey of asymptomatic schoolchildren and symptomatic patients.

Indeed, recruitment of symptomatic patients in sentinel centers is a convenient possibility for investigation and follow-up of malaria immune responses which is a mandatory public health structure that supports patients and relevant bio-clinical follow-up. However, this study showed clearly that active additional recruitment of asymptomatic individuals is needed for accurate analysis in such areas where malaria remains strongly endemic.

## Conclusion

This longitudinal immuno-epidemiological study conducted in peri-urban endemic setting of Abobo shows that immunity profiling using biomarker has the potential for immunological mapping of intervention and help for prevention measures towards malaria elimination. However, analysis of longitudinal variations of immunity in high endemic setting requires further studies delineating the relationship between seroprevalence and transmission intensity. The limited but useful snapshot picture of the high immune background from this study underlines the need for multiple sero-surveys in enlarged cohort of individual among populations in Côte d’Ivoire with different levels of exposure for accurate immune profiling.
